# The comparison of clinical outcome of fresh type II odontoid fracture treatment between anterior cannulated screws fixation and posterior instrumentation of C1-2 without fusion: a retrospective cohort study

**DOI:** 10.1186/s13018-017-0702-0

**Published:** 2018-01-08

**Authors:** Suomao Yuan, Bin Wei, Yonghao Tian, Jun Yan, Wanlong Xu, Lianlei Wang, Xinyu Liu

**Affiliations:** 1 Spine Center, Qilu Hospital of Shandong University, Wenhua West Road 107#, Jinan, 250012 People’s Republic of China; 2 Reproductive Medicine Centre, Maternal and Child Health Care Hospital of Shandong Province, Key Laboratory of Birth Regulation and Control Technology of National Health and Family Planning Commission of China, Jinan, 250014 People’s Republic of China

**Keywords:** Fresh, Odontoid fracture, Type II, Anterior, Posterior, Outcome

## Abstract

**Background:**

Recently, the excellent outcomes of temporary fixation of C1-2 without fusion in the treatment of odontoid fracture had been reported. It is still unclear if this technique could achieve the equivalent outcomes as the golden standard technique of anterior screw fixation. The objective of this study is to compare the clinical outcome of two treatments of fresh type II odontoid fracture: anterior cannulated screws fixation (ACSF) versus posterior instrumentation of C1-2 without fusion (PIWF).

**Methods:**

This is a retrospective study. This series included 28 males and 8 females, and the mean age was 41.5 years (range, 22 to 70 years). Eleven patients were treated with ACSF, and 25 patients with PIWF. For PIWF, the implants were removed after fracture union was confirmed at 0.75~1.5 years later. All patients underwent preoperative and serial postoperative clinical examinations at approximately 3 months, 6 months, and annually thereafter. The neck disability index (NDI) was used to assess the neck discomfort caused by the operation. The range of rotary motion was evaluated at each visit. All fractures were reassessed postoperatively with serial X-films and CT scans of the cervical spine at each follow-up visit, to evaluate screw position, fracture alignment, and fusion status.

**Results:**

All patients achieved immediate spinal stabilization after surgery, and none experienced neurologic deterioration. The follow-up periods ranged from 24 to 60 months. The average range of neck rotation was dramatically lost in PIWF after fixation (46° and 89° respectively in ACSF and PIWF), and recovered to 83° after the implant was removed. The NDI in PIWF was statistically higher than that in ACSF (5 and 13% respectively in ACSF and PIWF) after the first operation and decreased to 8% 1 year after the secondary operation. The fusion rates were 90.9 and 96% respectively in ACSF and PIWF. Both groups had a case of fracture non-union.

**Conclusions:**

For fresh type II odontoid fractures, high rate of fracture union can be achieved by both ACSF and PIWF. For most fresh type II odontoid fractures, anterior screw fixation was the best option for its simplicity and preservation of normal atlanto-axial rotary function. Posterior instrumentation without fusion could preserve most of the atlanto-axial rotary function and lead to moderate neck discomfort and is also a good alternative if anterior screw fixation is contraindicated.

## Background

Odontoid fractures are classified into three types according to fracture location in the sagittal plane (I, II, or III) [1]. Fractures of the dens comprise 18 to 20% of cervical injuries, of which 65 to 74% are Anderson-D’Alonzo type II fractures [2–4]. The common injury mechanism typically consists of high falls or traffic accidents. Atlanto-axial instability is common in type II odontoid fractures, and the treatment can be difficult. The treatment varies from conservative treatment with brace or halo-vest to a variety of surgical treatments. The treatment of type II odontoid fractures remains controversial since the nonunion rate is high for type II fractures, and the risk factors have been suggested. To acquire fracture union, immobilization is essential for these fractures. The difficulty is to determine how such immobilization should be achieved and maintained: collar immobilization halo immobilization, anterior or posterior internal fixation [5–8].

The reported union rate for type II fractures treated with immobilization in either a halo vest or a sternal occipital mandibular immobilizer brace was 76% [9]. The reported union rates of surgical treatment ranged from 88 to 100% [10–14]. Surgical treatment is indicated in patients with unstable fractures. Surgery is necessary to align and stabilize the upper cervical spine protecting the neural elements.

However, the selection of surgical management remains controversial, especially in the geriatric population. Currently, anterior screw fixation has been a popular surgical treatment. The reported success rates have been relatively high, averaging 94.5% with a low rate of implant-related complications and a very low rate of neurovascular injury [12, 15–18]. The rotation function can be well protected in anterior screw fixation. However, this procedure is contraindicated in some special odontoid fractures. Contraindications to this procedure include fracture comminution, an unfavorable fracture plane angulation from anterior caudal to posterior rostral, rupture of the transverse atlanto-axial ligament, and inability to obtain anatomic fracture reduction.

Posterior surgical stabilization has evolved from posterior wiring and clamps to transarticular screws and subsequent C1-C2 instrumentation with screw-plate and screw-rod system [17, 19, 20]. Fusion rate is low in wiring techniques, and the application of this technology is currently less. Transarticular screw fixation in conjunction with a wiring technique can acquire a union rate in excess of 95% [21]. Posterior fusion is typically accomplished with use of iliac crest structural autografts versus various onlay allografts.

The posterior procedure can provide strong fixation and acquire high rate of bone healing. However, the sacrifice of atlanto-axial rotary function is inevitable. To preserve the atlanto-axial rotary function, the simple fixation without fusion via posterior approach has been tried. Ma et al. reported the preliminary outcome of posterior nonfusion screw-rod fixation for preserving the atlanto-axial rotary function due to fresh type II odontoid fracture [22]. Their results showed all the odontoid fractures acquired bony healing and the atlanto-axial rotary function recovered to the normal 6~12 months after the instrumentation was removed. Han et al. reported their preliminary results of temporary pedicle screw fixation in 13 patients and they concluded temporary pedicle screw fixation is a feasible technique for motion preservation of type II odontoid fractures unsuitable for anterior screw [23]. Although these reports demonstrated excellent results, the outcome comparison between anterior screws fixation and posterior instrumentation without fusion has not been reported.

We designed a retrospective study to evaluate the bone union rate and neck rotary function of anterior cannulated screws fixation (ACSF) and posterior instrumentation of C1-2 without fusion (PIWF). The neck discomfort caused by the operations was also assessed.

## Methods

### Patients’ selection

This study was approved by the Ethics Committee, Qilu Hospital of Shandong University. A retrospective search for the patients who underwent anterior screw fixation or posterior atlanto-axial instrumentation without fusion for fresh type II odontoid fractures from March 2008 to March 2013 in our institution revealed that 36 patients met the inclusion criteria. This group included 28 males and 8 females, and the mean age was 41.5 years (range, 22 to 70 years). Eleven patients (8 males and 3 females) were treated with ACSF, and 25 patients (19 males and 6 females) with PIWF. Data for each patient were obtained from hospital medical records of clinical examination, operative findings, and imaging studies. All subjects had fractures from trauma and underwent radiographic examination via cervical spine radiography, CT and MRI prior to surgery. All fractures were assessed preoperatively by evaluating the initial lateral and open-mouth anteroposterior radiographs and CT scans of the odontoid process. The injury or compression of spinal cord was evaluated by MRI. Three experienced surgeons performed the surgical procedures, all the PIWF were performed by one of them, and the ACSF were performed by the other two surgeons. In the present study, all the PIWF procedures were performed by the same one surgeon L.X.Y, an experienced surgeon in our institution. The ACSF procedure is typically implicated with the type I and II fractures, however, as the surgeon L.X.Y has experienced serious complications twice on a continuum with the ACSF procedure in the early period (not included in the present study as it happened years ago), and since then he preferred the PIWF even in type I and II fracture patients. During the following years, he found the PIWF is a useful choice, and it is why he performed the study to provide evidence upon it.

### Surgical technique

Skull traction was performed before the operation. The general anesthesia was carried out with the continuous skull traction to avoid the fracture dislocation caused by the neck hyperextension. Protection of the spinal cord during intubation is paramount. According to the authors’ experience, the usage of skull traction and fibrolaryngoscope during the intubation can avoid the excessive hyperextension and fracture dislocation.

### Anterior cannulated screw fixation (ACSF)

**Fig. 1:**
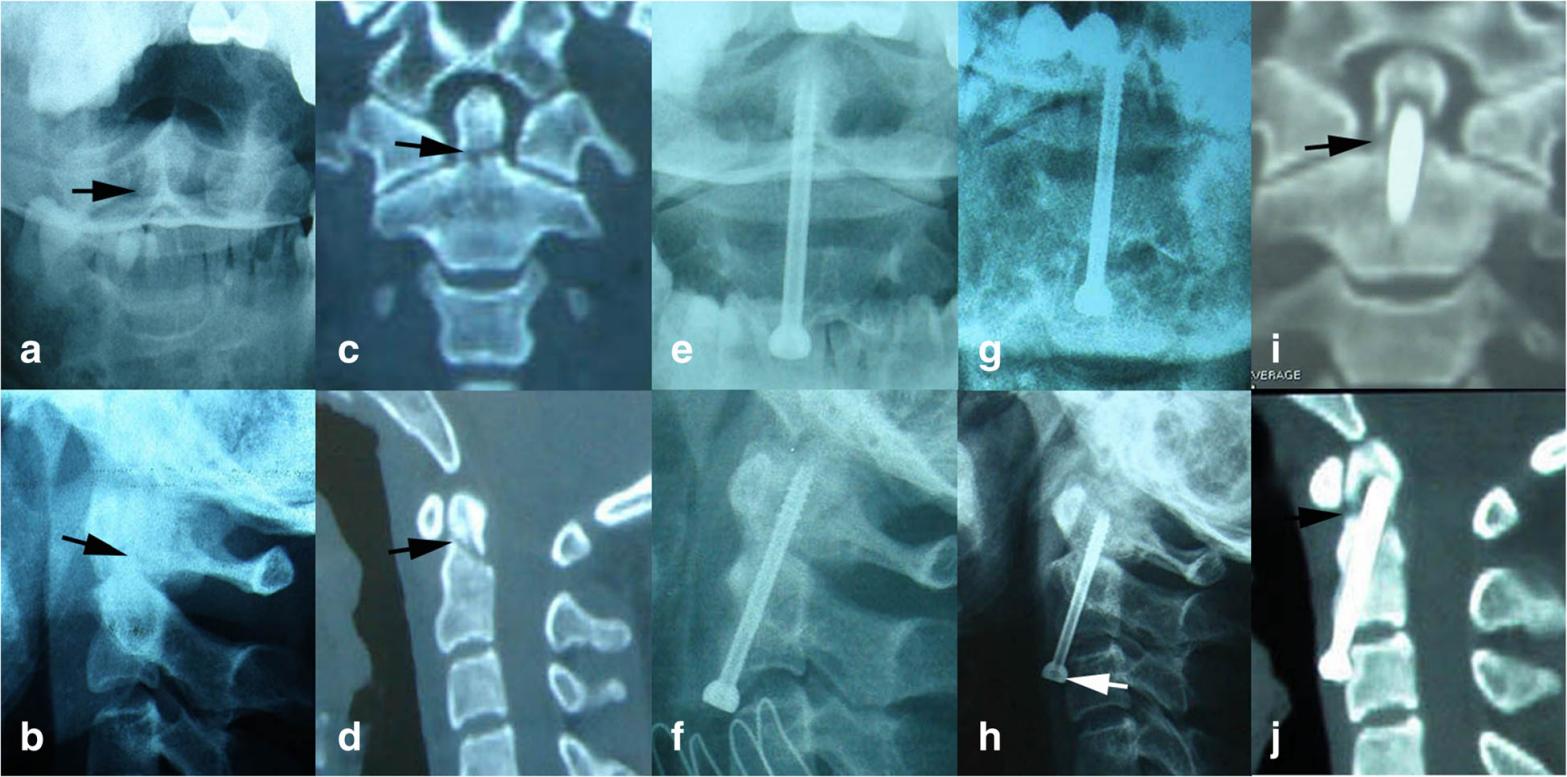
A failure case of ACSF. **a**, **b** Preoperative X-rays: *black arrows* indicate the fracture line. **c**, **d** Preoperative CT scans: the fracture line (*black arrows*) is clear in coronary and sagittal plane. **e**, **f** Postoperative cervical X-rays immediately after operation. **g**, **h** Postoperative X-rays 2 years after operation: the screw exits partly (*white arrow*). **i**, **j** Postoperative CT scans 2 years after operation: the fracture line (*black arrows*) is still visible and fracture nonunion is confirmed

Eleven patients were treated with ACSF. The patient was placed supine on the operating table, with his or her head fixed in a hyperextension position with skull traction. Firstly, the reduction of the fracture is attempted under the monitoring of fluoroscopy. Commonly, the fracture could be reduced easily with gentle traction and cervical extension. A transverse incision is made along the skin crease at the level of C5 to C6. A Smith–Robertson approach is used to expose the prevertebral space. The prevertebral fascia and longus colli muscles are elevated bilaterally to expose anterior–inferior C2 vertebral body. The entry point is in the middle of anterior–inferior edge of C2. This entry point should be slightly recessed in the anterior—most aspect of the C2 to C3 disc space to prepare a place for the burial of the screw head. A 2-mm K-wire is drilled into odontoid process under fluoroscopy. A 7-mm hollow hand drill is used to enlarge the trajectory over the K-wire. The apical cortex of the odontoid should be penetrated to achieve more purchase for the screw. The drilling must be meticulous and monitored by continuous fluoroscopy to avoid the potential injury of the spinal cord. The measurement of the length of the screw is a particularly import step in the whole operation. The lag effect, which is the action of pulling two fractured bony elements together, will be affected if the screw is too short or too long. A tap is inserted and used to tap the pilot hole to the apical cortex. A cannulated lag screw is inserted along the K-wire and advanced through the tapped hole (Fig. 1e, f). As the screw tightens, the distal fragment is pulled toward the proximal fragment. Finally, the head of the screw is then buried into the C2 body. We typically prefer a rigid collar postoperatively for 3 months.

### Posterior instrumentation of C1-2 without fusion (PIWF)

Twenty-five patients were treated with PIWF. Prone positioning can be performed on a well-padded operating table with large chest and pelvic rolls horizontally oriented. The rolling of the body after the anesthesia should be performed carefully with the aid of rigid collar and skull traction to avoid the aggregation of fracture displacement during the positioning. After positioning, the reduction of the fracture is attempted under the monitoring of fluoroscopy. For most cases, the reduction can be achieved by gentle traction and hyperextension. During the fracture reduction, the sustained skull traction can facilitate this process and avoid the accidental fracture dislocation. Restoration of the posterior vertebral line is assessed to ensure adequate reduction. The skin is marked over the location of the C1 ring and the caudal C2 spinous process.

**Fig. 2:**
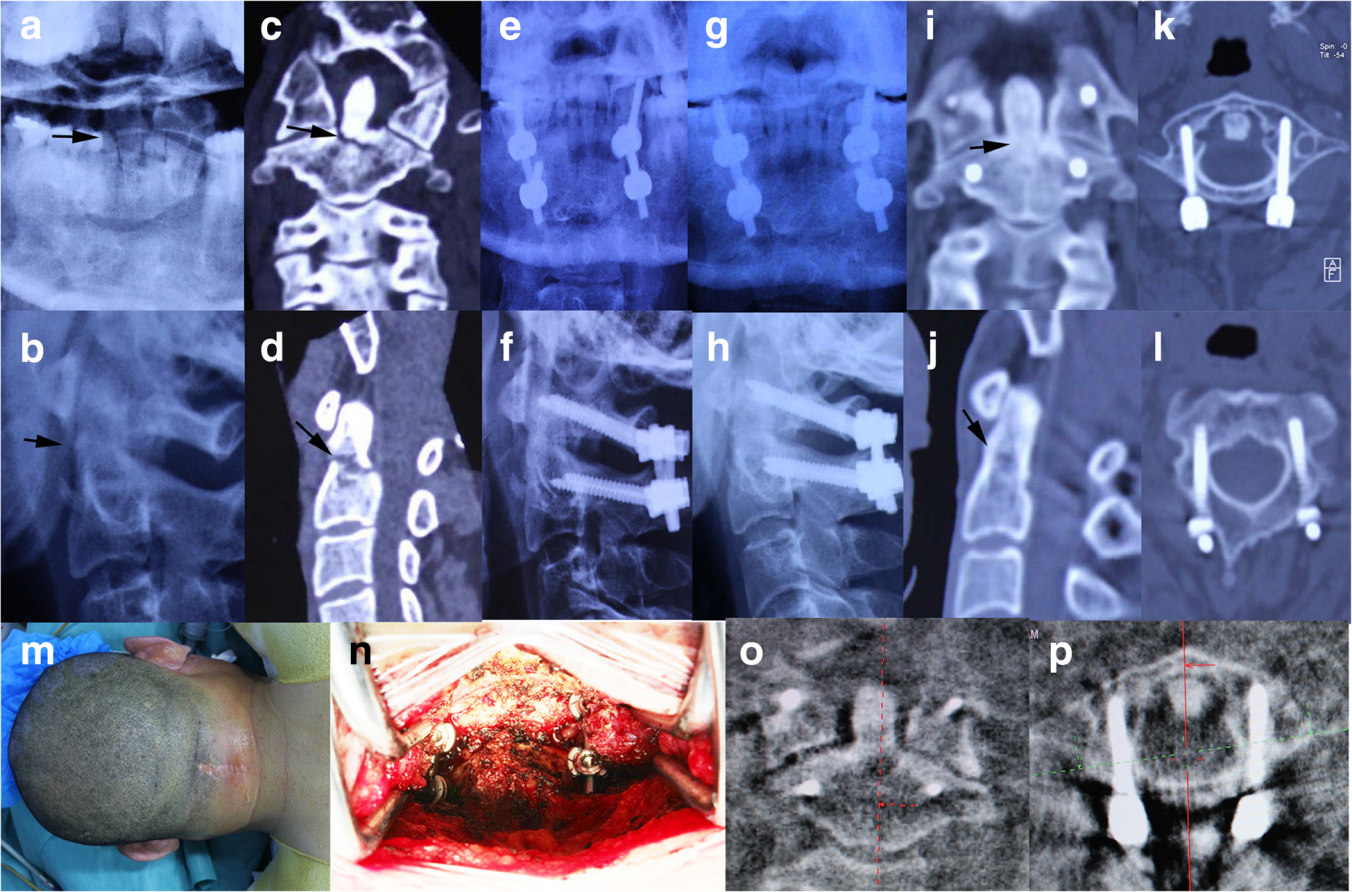
A successful case of PIWF. **a**, **b** Preoperative X-rays: *black arrows* indicate the fracture line. **c**, **d** Preoperative CT scans: the fracture line (*black arrows*) is clear in coronary and sagittal plane. **e**, **f** Postoperative X-rays immediately after operation. **g**, **h** Postoperative X-rays 1 year after operation. **i**–**l** CT scans 1 year after operation: the trabeculation (*black arrows*) across the fracture site confirms the bony union. **m** Skin incision. **n** Intraoperative picture demonstrating C1 lateral mass screws and C2 pedicle screws. **o**, **p** Intraoperative CT scans: the screws are placed in the accurate positions

A posterior midline incision about 4~5 cm is used (Fig. 2m). Dissection through the midline of the ligamentum nuchae will minimize the blood loss. The medial 10 mm of C1 ring and the cranial half of C2 spinous process are exposed with a combination of monopolar and bipolar cautery. The exposure then proceeds to the C2 lateral masses with care not to violate the C2-3 joints. With a Penfield elevator, the inferior half of posterior surface of the C1 ring is exposed carefully until the entry point of C1, which is measured preoperatively on the axial CT, is visualized. The cranial aspect of the C2 pedicle is palpated using a winged-tip elevator to determine the entry point of C2. The C1-2 posterior venous plexus is remained intact to avoid bleeding.

### C2 fixation

The entry point of C2 screw is determined by palpating the medial wall of the C2 pedicle with a Penfield elevator. A 2-mm burr is used to create a starting point. The screw path is directly in line with the visualized C2 dorsal pedicle. A series of hand drills with different lengths are used to create the trajectory along the pedicle direction. After each drilling, four walls are then palpated with a ball-tip feeler to make sure no wall is broken. The entry point or the drilling direction should be adjusted if any of the four walls is pierced, especially the medial wall. The cortex is much thicker laterally than medially which helps protect against a lateral breach and potential injury of vertebral artery. After tapping, a proper length of screw is placed bilaterally (Fig. 2e, f, n, o).

### C1 fixation

The C1 lateral mass screw starting point is on the posterior ring. This position is close to vertebral artery, and the risk of artery injury is high. Therefore, the starting point should be placed on the lower half of the posterior ring, which is close to the inferior edge of C1 ring, to maintain a safe distance to vertebral artery. Measurement of the width of each C1 lateral mass on the preoperative CT axial images aids in medial-lateral screw starting point selection and screw trajectory. The combination with 3D image of posterior aspect of atlas can help to determine the best position of starting point. If the starting point is difficult to determine by visual landmark, the palpation of medial edge of the C1 lateral mass is helpful to find the starting point, which is usually 4 mm lateral to medial edge. Once the position of starting point is confirmed, the assistant should use two Penfield elevators to protect the surrounding structure superiorly and laterally. A 2-mm burr is used to create the starting point. As described previously, the screw trajectory is prepared with hand drill. Under lateral fluoroscopy, the drilling should be pointed to the C1 anterior tubercle. Drilling is stopped once the posterior aspect of the C1 anterior tubercle is reached on lateral fluoroscopy. A ball-tipped feeler is used to palpate four walls of the trajectory to make sure there are no breaches. The screw length is then measured, and the screw is placed (Fig. 2e, f, n, o, p).

Bone grafting was not performed after internal fixation to facilitate the recovery of atlanto-axial rotary function after implant removal.

### Postoperative clinical evaluation and follow-up

All patients underwent serial postoperative clinical examinations at approximately 3 months, 6 months, and annually thereafter. The neck disability index (NDI) was used to assess the neck discomfort caused by the operation.

**Fig. 3:**
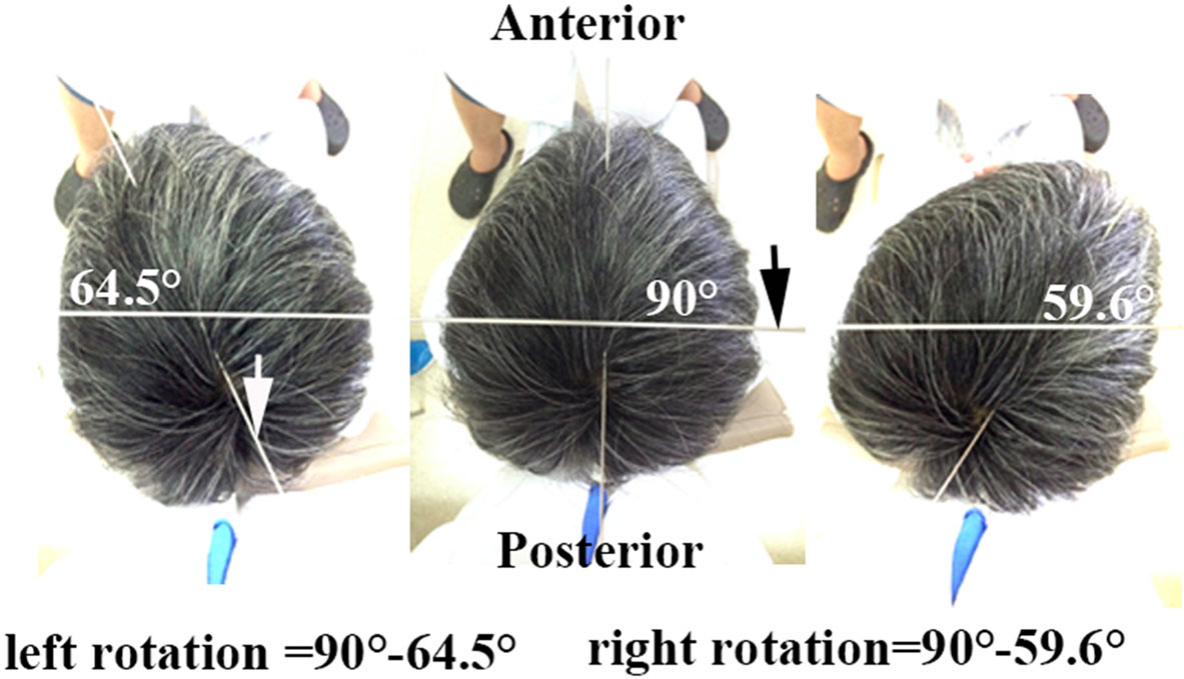
The measurement of the neck rotary motion. **a** The patient turns the head to the left to the unmost and the rotation angle is measured. **b** The patient sits on a chair with the head in the neutral position and the eyes straight ahead. One indicating device (*white arrow*) was fixed on the head. Another indicating device (*black arrow*) was fixed over the head. **c** The patient turns the head to the right to the unmost and the rotation angle is measured

The range of neck rotary motion was evaluated at each visit and calculated as the average degree of right and left rotation. The measurement of the neck rotary motion was performed with the patients sitting on a chair. One indicating device was fixed on his or her head. Another indicating device was fixed over the head. The patient rotated his/her neck from the neutral position to the maximum while keeping the shoulders still and the eyes horizontally. The pictures of the starting point and the ending point were captured. Then, the range of neck rotary motion of both sides was calculated (Fig. 3).

**Fig. 4:**
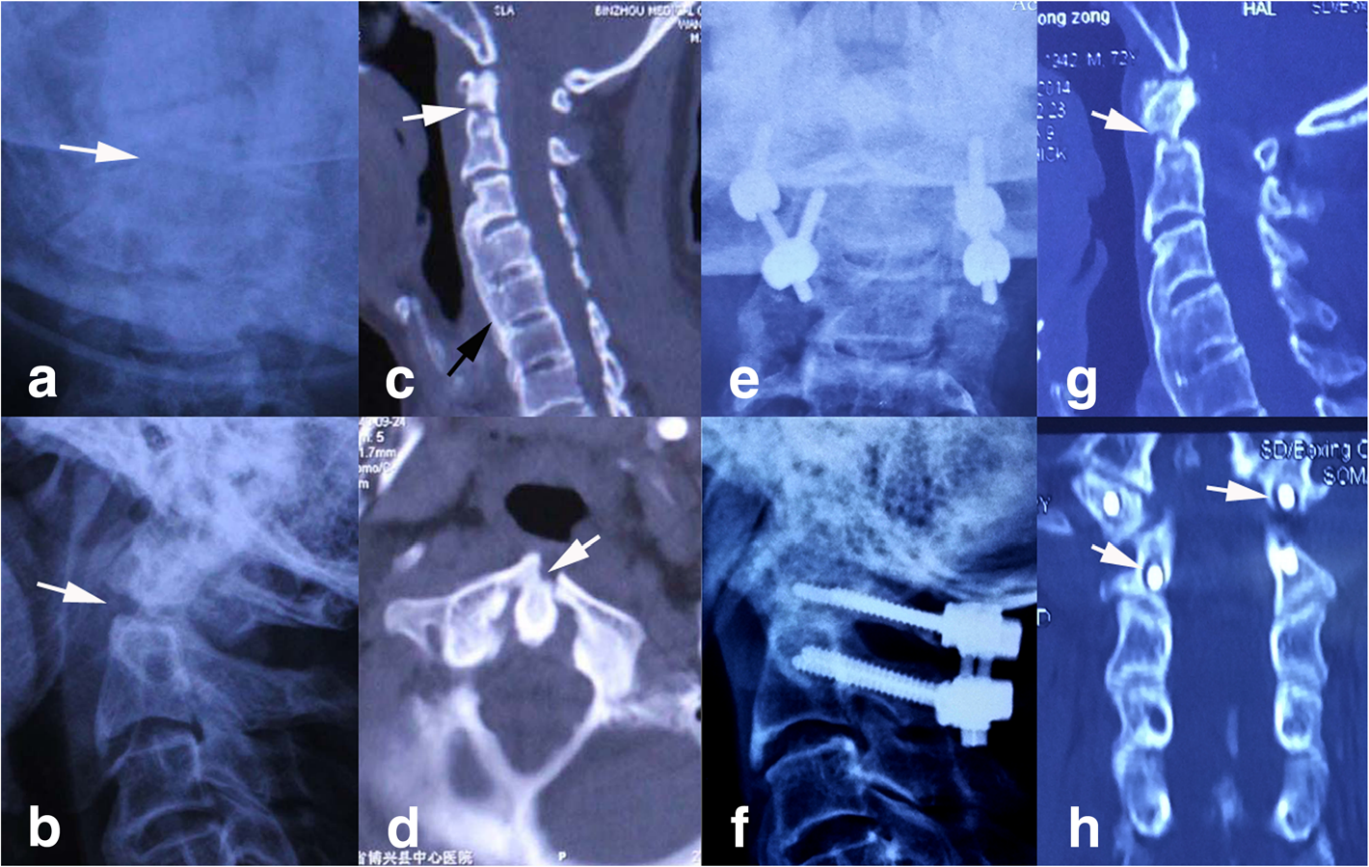
A failure case of PIWF. **a**, **b** Preoperative X-rays: *white arrows* indicate the fracture line. **c** Preoperative CT scans: the fracture line (*white arrow*) is clear in sagittal plane and the extensive fusion (*black arrow*)of C3-7 confirms the diagnosis of diffuse idiopathic skeletal hyperostosis (DISH). **d** Preoperative CT scans: fracture of anterior arch of atlas (*white arrow*). **e**, **f** Postoperative X-rays immediately after operation demonstrate ideal fragment reduction after PIWF. **g**, **h** Postoperative CT scans 3 years after operation shows the fracture failed to unite and the screws are loose

All fractures were reassessed postoperatively with serial X-films and CT scans of the cervical spine at each follow-up visit, to evaluate screw position, fracture alignment, and fusion status. Bone fusion was considered successful if solid bony fusion, seen as trabeculation across the fracture site, was present on both X-films and sagittal and coronary reconstruction of cervical CT scans (Fig. 2g–j). Unsuccessful fusion (nonunion or fibrous union) was determined when the fracture line was visible on lateral X-ray studies or sagittal and coronary reconstruction of cervical CT scans (Figs. 1g–j and 4g).

For the patients undergoing PIWF, the implant was removed 0.75~1.5 years later once the bony fusion of fracture was confirmed. After the secondary operation, the patients were followed up for at least 1 year. The NDI and range of neck rotary motion were evaluated at each visit.

### Statistical analysis

Because of the small sample size, the two groups were compared with the Student’s *t* test for continuous variables and the Fisher’s exact test for categorical variables. All statistical assessments were two-sided and evaluated at the 0.05 level of significant difference. Statistical analyses were performed using SPSS 20.0 statistics software (SPSS Inc., Chicago, IL, USA).

## Results

There were 28 males and 8 females. The mean age was 42 years (range 20–66 years) in ACSF, including 5 cases (45.5%) less than 40 years old, 3 cases (27.3%) of 40–60 years old, and 3 cases (27.3%) of more than 60 years old. Of the 11 patients, 5 (45.5%) had fractures sustained in motor vehicle accidents and six (54.4%) had fractures resulting from falls. The average time interval from injury to operation was 5 days (range 0.2–11 days) (Table 1).

**Table 1:** Characteristics of 36 patients with fresh type II odontoid fracture who underwent ACSF or PIWF

Characteristic	ACSF (*n* = 11)	PIWF (*n* = 25)	*p*
Age, years, mean	42(20–66)	42(22–70)	0.957
< 40 years, *n*(%)	5(45.5)	9(36)	0.022
40–60 years, *n*(%)	3(27.3)	14(56)	
> 60 years, *n*(%)	3(27.3)	2(8)	
Gender, *n* (%)			
Female	3(27.3)	6(24)	0.571
Male	8(72.7)	19(76)	
Causes of fractures, *n*(%)			
Traffic accident	5(45.5)	9(36)	0.716
Falls	6(54.5)	16(64)	
Grauer type, *n*(%)			
IIA	6(54.5)	11(44)	0.002
IIB	5(45.5)	9(36)	
IIC	0(0)	6(24)	
Associated spinal cord injury, *n*(%)	3(27.3)	8(32)	0.002
Associated other injuries, *n*(%)	5(45.5)	14(56)	1.000
Interval from injury to operation, mean days (range)	5(0.2,11)	3.2(0.2,8)	0.163
Follow-up duration (months)	42.1	43.1	0.226

*ACSF* anterior cannulated screw fixation, *PIWF* posterior instrumentation of C1-2 without fusion

The mean age was 42 years (range 22–70 years) in PIWF, including 9 cases (36%) less than 40 years old, 14 cases (56%) of 40–60 years old, and 2 cases (8%) of more than 60 years old. Of the 25 patients, 9 (36%) had fractures sustained in motor vehicle accidents and 16 (64%) had fractures resulting from falls. The average time interval from injury to operation was 4.6 days (range 0.4–10 days). According to Grauer classification, the fractures were type IIA in 6 cases and type IIB in 5 cases in ACSF, and type IIA in 11 cases, type IIB in 9 cases, and type IIC in 6 cases in PIWF. The associated spinal cord injury occurred in 3 cases (27.3%) in ACSF and 5 cases (32%) in PIWF. The associated other injuries, such as other cervical fracture (Fig. 4d), limb fracture, and craniocerebral injury, occurred in 5 cases (45.5%) in ACSF and 14 cases (56%) in PIWF. The mean follow-up period was 42.1 and 43.1 months with a minimum period of 2 years (range, 24–60 months) in ACSF and PIWF respectively (Table 1).

The average operation time and blood loss were 2.8 h and 37 ml in ACSF and 3.6 h and 198 ml in PIWF. Patients had minimal blood loss and experienced no complications such as bleeding, hoarseness, infection, or vascular or neural structure injuries in both groups. However, the blood loss in ACSF and PIWF was statistically different (*p* = 0.002).

All patients achieved immediate spinal stabilization after surgery, and none experienced neurologic deterioration. The average NDI was 5% in ACSF and 13% in PIWF at 1-year follow-up after the first operation and was 4% in ACSF and 8% in PIWF at 1-year follow-up after the secondary operation. The union rate was 90.9% in ACSF and 96% in PIWF (Table 2).

**Table 2:** Clinical outcomes of 36 patients with fresh type II odontoid fracture who underwent ACSF or PIWF

Parameter	ACSF (*n* = 11)	PIWF (*n* = 25)	*p*
Average operation time (minutes)	121	173	0.007
Average blood loss (ml)	33	148	0.002
Complication, *n*(%)	0(0)	0(0)	
Average NDI (%)			
1 year after first operation	5	13	0.000
1 year after second operation	4	8	0.009
Average range of rotatory motion (°)			
1 year after first operation	89(83,93)	46(35,58)	0.000
1 year after second operation	88(81,95)	83(79,87)	0.148
Union rate (%)	90.9	96	0.524

Patient age, gender, cause of fracture, and time interval from injury to operation were not statistically significant between two groups. The neck discomfort caused by PIWF was more serious than that by ACSF (*p* = 0.000). ACSF had little impact on the neck rotary motion; however, PIWF could significantly reduce the neck range (*p* = 0.000).

After the implant was removed, the neck discomfort was significantly reduced in PIWF while it was still more serious than in ACSF (*p* = 0.009). Meanwhile, the neck rotary motion in PIWF was recovered apparently and was close to that in ACSF (*p* = 0.148).

The fusion rates were 90.9 and 96% in ACSF and PIWF respectively, and the difference was not statistically significant (*p* = 0.524). Both groups had a case of fracture non-union (Figs. 1 and 4). The ages were 32 years (ACSF) and 70 years (PIWF) respectively. The only complaint was moderate neck discomfort, and both of them refused to undergo a secondary surgery. Conservative treatments including brace application, avoiding strenuous activities, and muscular training were utilized. The follow-up periods were 24 months (ACSF) and 36 months (PIWF) respectively. The neck discomfort did not relieve or aggravate, and no other morbidity occurred during the follow-up. In addition, implant failure did not occur in the two patients who had unsuccessful fusion at the last follow-up, maybe due to the formation of the fibrocartilage linkage between the interfragmental gap.

## Discussion

Anderson and D’Alonzo classified odontoid fractures as type I, II, or III [24, 25]. Type II fractures have a watershed blood supply and have a high nonunion rate with risk for subsequent chronic pain, atlanto-axial instability, and neurological deterioration along with high mortality rates [26]. Hadley et al. [27] identified an additional fracture subtype (IIA) based on comminution of the odontoid base with decreased stability. These classifications were later expanded upon by Grauer, who included three different subtypes of type II fractures based on displacement, comminution, and fracture line obliquity to help guide treatment [28]. A type IIA fracture describes a minimally or non-displaced fracture without comminution which may be treated with external immobilization. A type IIB fracture line extends craniocaudal anterior-superior to posterior-inferior and is amenable to anterior screw osteosynthesis. Type IIC fracture extends caudocranially from anterior-inferior to posterior-superior with or without comminution. This fracture type is more amenable to posterior C1-2 fusion. However, identifying the optimal treatment for a type II fracture has been difficult. There are many factors that should be considered to choose the optimal treatment of type II fractures, such as patient age, type of fracture, extent and direction of fracture displacement, associated other injuries, and potential possibility of fracture fusion. Because the instability of these fractures places patients at significant risk for further disastrous spinal cord injury, the fracture stabilization should be acquired as early as possible. However, the treatment of odontoid fracture can be challenging because of the complex anatomy of C1 and C2. The type II fracture occurs through the base of the odontoid process and the blood supply to the odontoid process may be compromised.

Currently, ACSF has been a popular surgical treatment. This technique was first reported in 1980 by Nakanishi [16]. Numerous studies have reported the high fusion rate of anterior screw fixation to stabilize type II odontoid fractures [12, 13, 17, 29–31]. Hou et al. [29] reported that anterior odontoid screw fixation is an effective approach for direct fracture fixation without autologous bone graft. These researchers cited a number of advantages over posterior C1 arthrodesis, including immediate stabilization, less postoperative pain, no requirement for bone graft, and preservation of the normal atlanto-axial rotational movement. This was true despite the fact that their patients had a mean age of 80 years. It has been proved that anterior screw fixation can be a safe option for elderly patients with type II odontoid fracture. High fusion rates, low postoperative complications, and maintenance of cervical rotary motion can be achieved. These are also confirmed in our study. Our results showed the fusion rate is as high as 90.9% in ACSF, and no postoperative complication occurred. ACSF had little impact on the neck rotary motion.

The success of this treatment depends on patient selection, attention to technical operative details, and adequate follow-up. Internal screw fixation gives immediate direct fixation of the fracture, offers a high rate of fusion without requiring prolonged halo/vest immobilization, reduces occurrence of the cervical pain, and preserves the normal mobility of C1-C2 [32]. In acute axis fractures, early surgical intervention within 3–5 days of injury is recommended for patients exhibiting acute fracture instability despite external immobilization, transverse ligament disruption, or type II odontoid fractures with dens displacement of at least 6 mm on admission [33]. In our study, the union rate of 90.9% is comparable to other reports, in which the success rate has ranged from 73 to 100%.

Fractures that pass from anteroinferior to posterosuperior (anterior oblique orientation), which is described as type IIC in Grauer’s research [28], are significantly more likely to result in non-anatomical union, non-union, or fibrous union than are posterior oblique and horizontally oriented fractures [34]. Aebi and colleagues concluded that an oblique fracture oriented from anteroinferior to posterosuperior provides only a small part of the C2 body for purchasing of the screw, which may result in anterior displacement of the fragment under compression [16]. Therefore, anterior oblique orientation is considered a contraindication to the anterior screw fixation technique, and proper fracture identification and selection are keys to success [35, 36].

However, the patient that failed to acquire bony union in ACSF in this study had a fracture with a posterior oblique orientation (type IIB) (Fig. 1d), for which the anterior screw fixation is recommended by Grauer [28]. Therefore, the nonunion was not a problem caused by the wrong selection of surgical procedure. Radiologic imaging of this patient who developed nonunion reveals the threaded portion of the screw impinged upon the fracture line (Fig. 1e, f), failing to produce a proper lag effect. The lag screw is designed with a threaded distal portion, a smooth proximal portion, and a wide head to simultaneously re-approximate the proximal and distal fragments during the screw tightening and to achieve compression fixation. To achieve the lag effect, the threaded portion must be engaged only in the distal fragment. If the threaded portion is too long and enters the fracture line, compression across the fracture line will not be possible. Several other reasons may also play a role to the failure of bony fusion in this patient. The size of the fracture fragment was small, and the effective purchase of the screw was limited. The apical cortex of the odontoid is failed to be penetrated, and this prevents the screw from obtaining more purchase. In addition, the selection of improper length of screw leads to the separation of two fracture fragments when the screw is tightened. These factors stress the importance of cortex penetration and proper selection of screw length when selecting patients for anterior screw fixation. This patient subsequently received conservative immobilization treatment and did not undergo further surgery because C1-C2 arthrodesis would have compromised her neck rotation by 50%, which the patient viewed as unacceptable. This patient received physical therapy and rehabilitation to increase their neck strength and did not request further surgical intervention because the neck pain was tolerable for her.

Posterior C1-2 arthrodesis is an alternative option for those special odontoid fractures for which anterior screw fixation is not suitable. Anderson et al. recommend first-line C1-C2 arthrodesis, due to the rates of complications and of non-union that are lower than with anterior screw fixation [37]. Traditionally, atlanto-axial instability has been treated by posterior wire stabilization and structural bone grafting using, for example, the Gallie or Brooks techniques [38]. Although these approaches are reasonably safe and effective, they limit primarily flexion and extension. Additionally, these methods do not provide appropriate stability in axial rotation or translation [39, 40], which results in nonunion rates of up to 30% even with halo vest immobilization [10, 41]. In 1986, Magerl and Seemann reported posterior C1-2 transarticular screw fixation for patients with atlanto-axial instability [42]. Transarticular screw fixation is biomechanically more rigid than sublaminar wiring and prevents the rotation and translation of the atlanto-axial complex and provides immediate multidirectional stability with a fusion rate of over 90%.

In 2001, Harms and Melcher described their technique used to achieve posterior atlanto-axial stabilization with polyaxial screw-rod system [19]. They achieved C1-C2 bony fusion in 100% of cases. Biomechanically, the overall rigidity achieved using the C1 lateral mass and C2 pedicle screws is similar to that achieved with transarticular screws. Jeon et al. reported a series of 17 patients treated with polyaxial screw-rod system and they achieved C1-C2 bony union in 94.1% of the series [43].

However, no matter what technique is used in posterior C1-2 arthrodesis, the compromise of the atlanto-axial rotary function is inevitable because the bone fusion is prerequisite. Ma et al. reported their trial to treat fresh type II odontoid fracture with polyaxial screw-rod system. In their research, the traditional bony fusion of C1-C2 was canceled to preserve the atlanto-axial rotary function and the results demonstrated that all the fractures achieved bony fusion. After a secondary operation to remove the implant, the atlanto-axial rotary function almost recovered to the normal. In our study, we found the neck rotary function was almost recovered to the normal after the implant was removed. The neck disability caused by the first posterior operation was obvious. Although the neck disability was relieved a lot after the implant was removed, the result was still worse than that in ACSF.

One patient in PIWF failed to achieve fracture union in our study. This patient was 70 years old, and the neck was rigid because of the associated diffuse idiopathic skeletal hyperostosis (DISH) (Fig. 4c). This patient was immobilized with Philadelphia collar for only half month for the intolerance. Patient’s age can also be a factor, contributing to nonunion in 30–50% of older patients in literatures [34]. Therefore, posterior C1-C2 arthrodesis might be the best option for the geriatric patients with rigid neck.

Low rates of complications have been reported during most case series of C1-2 posterior polyaxial screw-rod fixation techniques. Intraoperative complications are rare with careful preoperative planning and meticulous surgical technique. Potential intraoperative complications include spinal cord, brainstem, or cerebellar injury directly or indirectly via compression during reduction maneuvers or vascular injury due to inadvertent screw preparation and placement. Vertebral artery injury is the most feared complication. The vertebral artery is at risk during exposure of the C1 lateral ring and during C1 instrumentation, and C2 pedicle (lateral breach) screw placement. In our series, none of these intraoperative complications occurred.

There are some limitations in our study. First, the small size of the sample makes us give up the planned grouping according to the ages. As we know, age is an important factor affecting the fusion. In the future study, we will expand the sample and compare the outcomes of different ages. Second, the selection bias of the present retrospective study cannot be ignored as the selection of surgery patient is depended on the experience and habits of different surgeons. The patients were not randomly or double blinded selected and the surgery skills and perioperative treatment differs between the groups. However, though the selection bias is not ignorable, it is also not so huge that may influence the conclusions significantly. The base line characteristics are comparable between the groups, all the surgeons are experienced enough, the perioperative treatments are almost same in the same institution, and the reported outcomes are similar with the others. Third, as there is no type IIC fractures in the ACSF group (type IIC fractures were only treated with PIWF), which is more serious than the other two types, it may influence the selection bias significantly. Maybe, we should redefine the PIWF group as only including the type IIA and B fractures, though the present study has already proved the PIWF procedure is similar with the ACSF. Future studies of randomized double-blind trial with more strict design and large participations should be performed to compare the two procedures.

## Conclusion

For fresh type II odontoid fractures, high rate of fracture union can be achieved by both ACSF and PIWF. Low rate of intraoperative complications was observed in both two procedures. For most fresh type II odontoid fractures, ACSF might be the best option for its simplicity and preservation of normal atlanto-axial rotary function. PIWF is also a good alternative if ACSF is contraindicated. It is not necessary to sacrifice the atlanto-axial rotary function to ensure the acquirement of bony healing. PIWF has allowed for high fracture fusion rates, low complications, and low revision rates with excellent predictable outcomes in patients with unstable odontoid fractures. However, PIWF still leads to transient loss of the atlanto-axial rotary function, and the neck disability caused by two operations in PIWF is worse than that in ACSF.

## Abbreviations

ACSF: Anterior cannulated screws fixation
DISH: Diffuse idiopathic skeletal hyperostosis
NDI: Neck disability index
PIWF: Posterior instrumentation of C1-2 without fusion
